# Supplementary material for: Clozapine Worsens Glucose Intolerance, Nonalcoholic Fatty Liver Disease, Kidney Damage, and Retinal Injury and Increases Renal Reactive Oxygen Species Production and Chromium Loss in Obese Mice
Source: Int J Mol Sci. 2021 Jun 22;22(13):6680. doi: 10.3390/ijms22136680 (PMC8268139; doi:10.3390/ijms22136680)
Supplement: Supplementary file 1 [file ijms-22-06680-s001.zip › ijms-1220289-supplementary.pdf]

International Journal of
Molecular Sciences

MDPI

Article

Clozapine Worsens Glucose Intolerance, Nonalcoholic Fatty Liver Disease, Kidney Damage, and Retinal Injury and Increases Renal Reactive Oxygen Species Production and Chromium Loss in Obese Mice

Geng-Ruei Chang [1,†], Hsien-Yueh Liu [2,†], Wei-Cheng Yang [3], Chao-Min Wang [1], Ching-Fen Wu [1], Jen-Wei Lin [2], Wei-Li Lin [2,4], Yu-Chen Wang [5-8], Tzu-Chun Lin [1], Huei-Jyuan Liao [1], Po-Hsun Hou [9,10,*], Chee-Hong Chan [11,*], and Chuen-Fu Lin [12,*]

[1] Department of Veterinary Medicine, National Chiayi University, 580 Xinmin Road, Chiayi 60054, Taiwan; grchang@mail.ncyu.edu.tw (G.R.C.); leowang@mail.ncyu.edu.tw (C.M.W.)
[2] Bachelor Degree Program in Animal Healthcare, Hungkuang University, 6 Section, 1018 Taiwan Boulevard, Shalu District, Taichung 433304, Taiwan; lhy_vet@hk.edu.tw (H.Y.L.);
[3] School of Veterinary Medicine, National Taiwan University, 4 Section, 1 Roosevelt Road, Taipei 10617, Taiwan; yangweicheng@ntu.edu.tw (W.C.Y.)
[4] General Education Center, Chaoyang University of Technology, 168 Jifeng Eastern Road, Taichung 413310, Taiwan; (W.L.L.)
[5] Division of Cardiology, Asia University Hospital, 222 Fuxin Road, Wufeng District, Taichung 41354, Taiwan; richard925068@gmail.com (Y.C.W.)
[6] Department of Medical Laboratory Science and Biotechnology, Asia University, 500 Lioufeng Road, Wufeng District, Taichung 41354, Taiwan; richard925068@gmail.com (Y.C.W.)
[7] Division of Cardiovascular Medicine, China Medical University Hospital, 2 Yude Road, North District, Taichung 404332, Taiwan; richard925068@gmail.com (Y.C.W.)
[8] College of Medicine, China Medical University, 91 Hsueh-Shih Road, North District, Taichung 404333, Taiwan; richard925068@gmail.com (Y.C.W.)
[9] Department of Psychiatry, Taichung Veterans General Hospital, 4 Section, 1650 Taiwan Boulevard, Taichung 40705, Taiwan;
[10] Faculty of Medicine, National Yang-Ming University, 2 Section, 155 Linong Street, Beitou District, Taipei 11221, Taiwan; (P.H.H.)
[11] Division of Nephrology, Chang Bing Show Chwan Memorial Hospital, 6 Lugong Road, Lukang Township, Changhua 505029, Taiwan; cheehong.chan@gmail.com (C.H.C.)
[12] Department of Veterinary Medicine, College of Veterinary Medicine, National Pingtung University of Science and Technology, 1, Shuefu Road, Neipu, Pingtung 912301, Taiwan; cflin2283@mail.npust.edu.tw (C.F.L.)
† These authors contributed equally to this work.
* Correspondence: Tel.: +886-4-23592525 (P.H.H.); +886-975-617071 (C.H.C.); +886-8-7703202 (C.F.L.)

Citation: Chang, G.-R.; Liu, H.-Y.; Yang, W.-C.; Wang, C.-M.; Wu, C.-F.; Lin, J.-W.; Lin, W.-L.; Wang, Y.-C.; Lin, T.-C.; Liao, H.-J.; et al. Clozapine Worsens Glucose Intolerance, Nonalcoholic Fatty Liver Disease, Kidney Damage and Retinal Injury and Increases Renal Reactive Oxygen Species Production and Chromium Loss in Obese Mice. *Int. J. Mol. Sci.* **2021**, *22*, 6680. https://doi.org/10.3390/ijms22136680

Academic Editor: Abdelkrim Hmadcha

Received: 28 April 2021
Accepted: 18 June 2021
Published: 22 June 2021

Publisher's Note: MDPI stays neutral with regard to jurisdictional claims in published maps and institutional affiliations.

Copyright: © 2021 by the author. Licensee MDPI, Basel, Switzerland. This article is an open access article distributed under the terms and conditions of the Creative Commons Attribution (CC BY) license (http://creativecommons.org/licenses/by/4.0/).

Supplementary Figures.

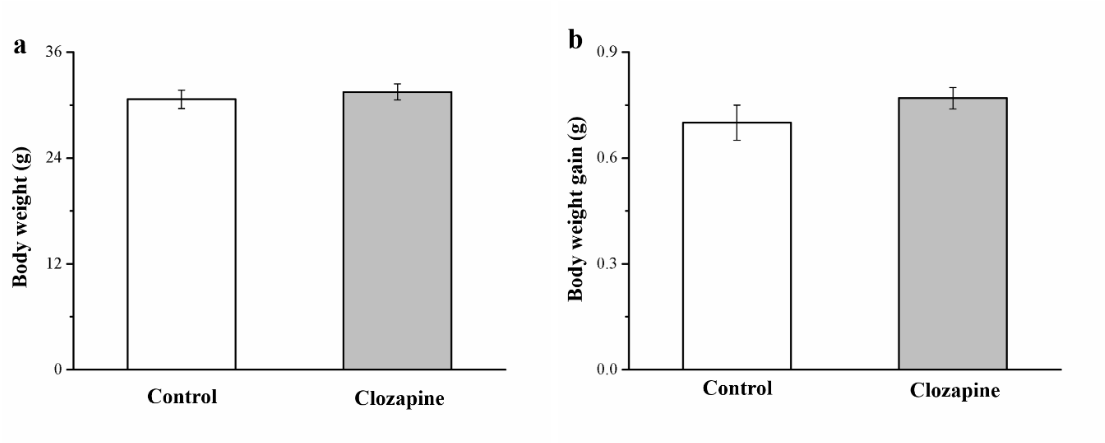

Figure S1. Effects of clozapine (2 mg/kg/day) on body weight and weekly weight gain measured in mice fed a standard diet and receiving control and clozapine treatment for 8 weeks.

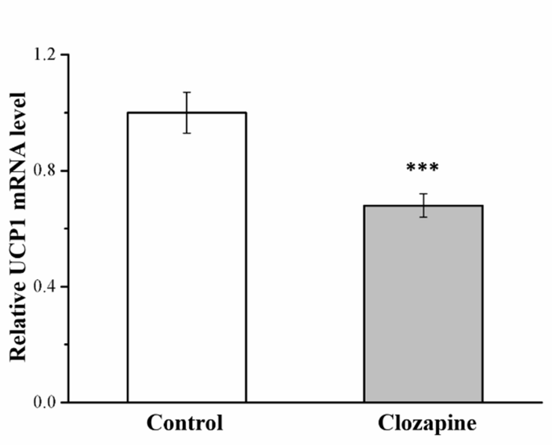

Figure S2. UCP1 mRNA expression measured in brown adipose tissue from the control and clozapine groups (all fed an HFD). ***$p < 0.001$.

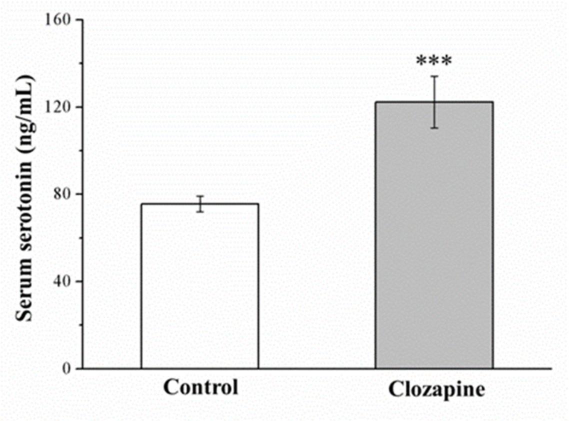

Figure S3. Serum levels of serotonin determined for the control and clozapine (receiving 2 mg/kg/day) groups. ***$p < 0.001$.

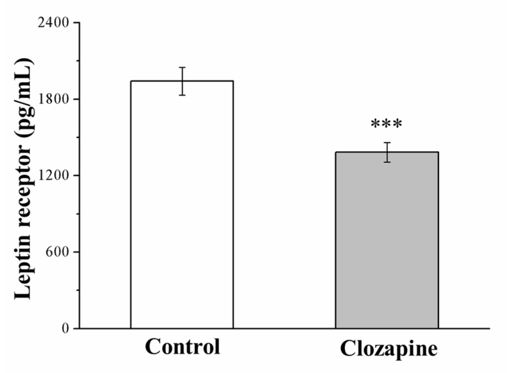

Figure S4. Serum levels of soluble leptin receptor determined for the control and clozapine (receiving 2 mg/kg/day) groups. *** $p < 0.001$.

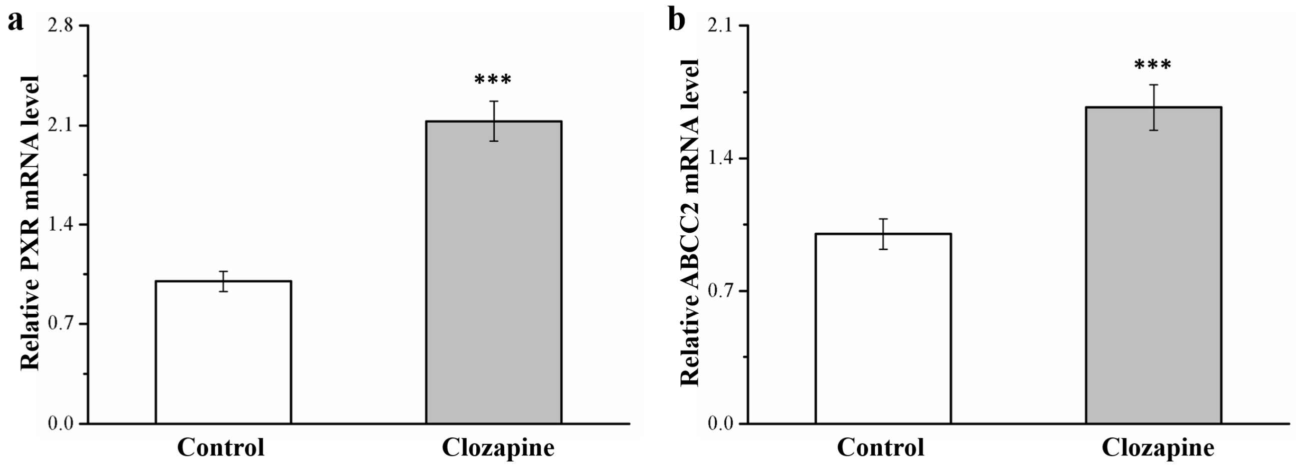

Figure S5. (a) *PXR* and (b) *ABCC2* mRNA levels in the livers determined for the control and clozapine (receiving 2 mg/kg/day) groups. *** $p < 0.001$.

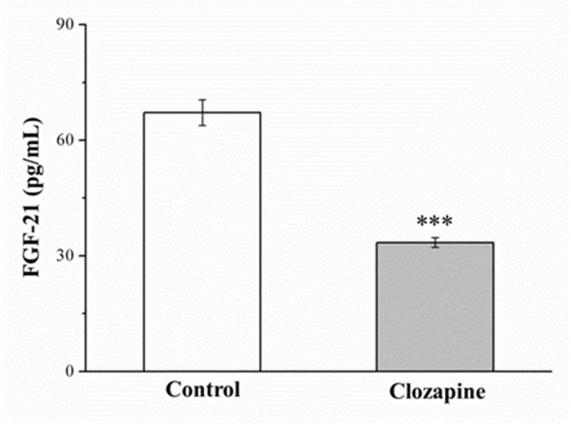

Figure S6. Serum levels of FGF-21 determined for the control and clozapine (receiving 2 mg/kg/day) groups. *** $p < 0.001$.

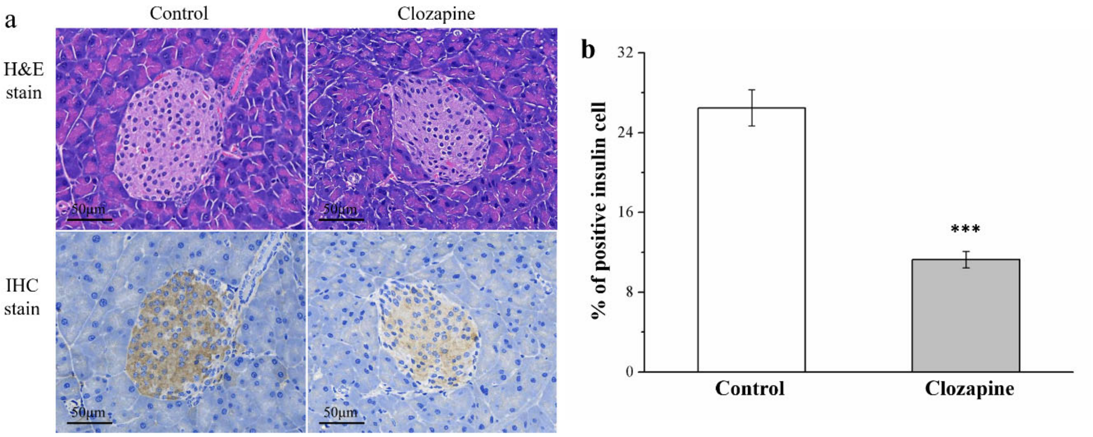

Figure S7. (a) H&E and immunohistochemical stains (magnification, 200×) showing islet morphology and (b) β-cell percentage of the control and clozapine (receiving 2 mg/kg/day) groups. ***$p < 0.001$.

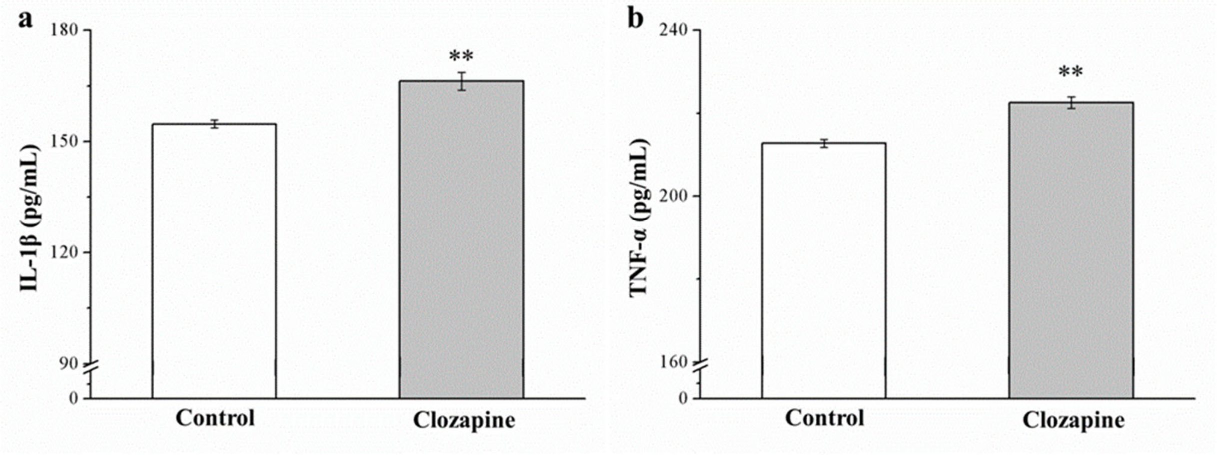

Figure S8. Serum levels of (a) TNF-α and (b) IL-1β determined for the control and clozapine (receiving 2 mg/kg/day) groups. **$p < 0.01$.

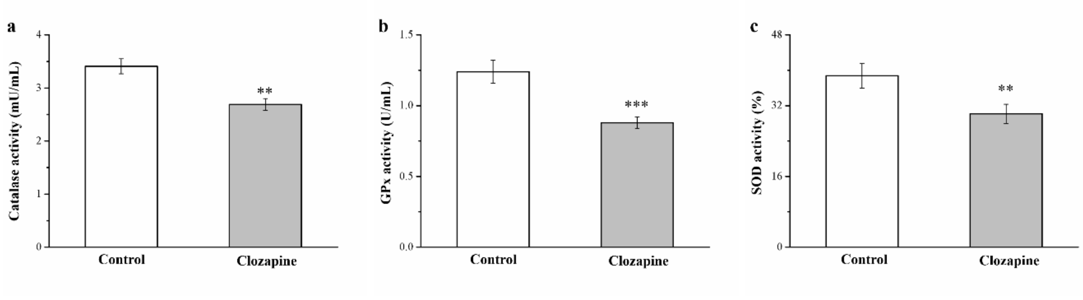

Figure S9. Pancreatic (a) catalase, (b) GPx, and (c) SOD activities in the control and clozapine (receiving 2 mg/kg/day) groups. **$p < 0.01$ and ***$p < 0.001$.

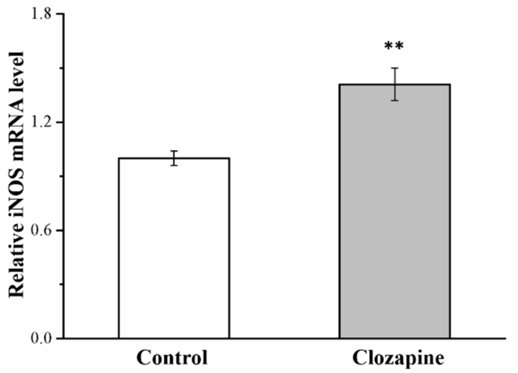

Figure S10. *iNOS* mRNA levels in the renal determined for the control and clozapine (receiving 2 mg/kg/day) groups. $^{**}p < 0.01$.

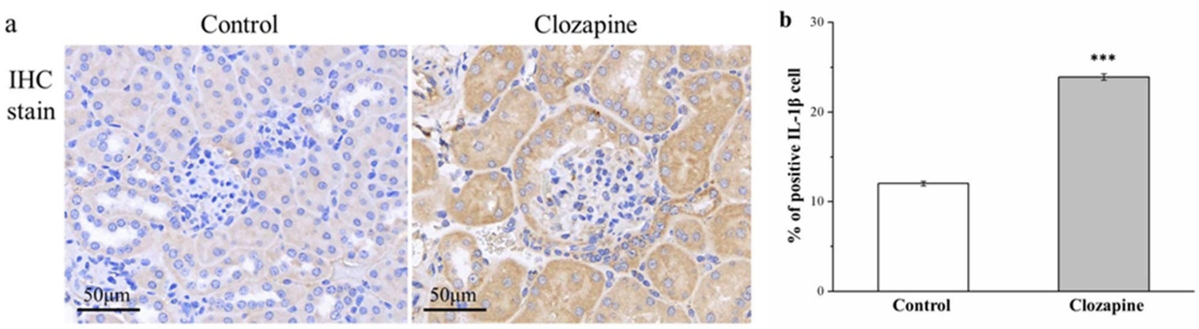

Figure S11. (a) Immunohistochemical stain (magnification, 200×) and (b) renal IL-1β levels of the control and clozapine (receiving 2 mg/kg/day) groups. $^{***}p < 0.001$.

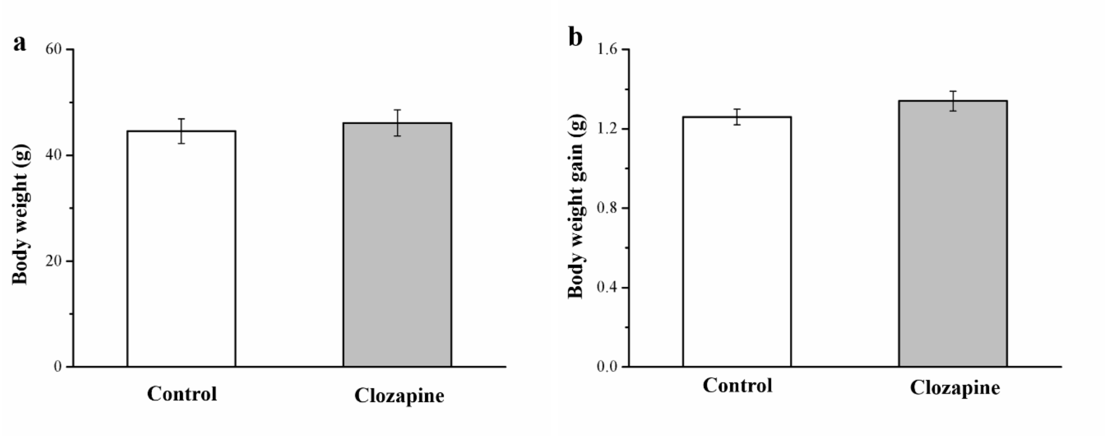

Figure S12. (a) Body weight and (b) weekly weight gain determined for the control and clozapine (receiving 1 mg/kg/day) groups.
